# Hepatitis A Leading to Severe Vitamin A Deficiency and Bitot's Spots in a Three-Year-Old Male Child: A Case Report

**DOI:** 10.7759/cureus.51821

**Published:** 2024-01-07

**Authors:** Nagaraju Nimmanagoti, Ashish Varma, Amar Taksande, Revat J Meshram

**Affiliations:** 1 Pediatrics, Jawaharlal Nehru Medical College, Wardha, IND

**Keywords:** tertiary care center, abdominal distension, pediatric patient, bitot's spots, vitamin a deficiency, hepatitis

## Abstract

This case presentation details the clinical journey of a three-year-old male child presenting with fever, abdominal distention, and loose stools. The child's symptoms, unresponsive to initial treatment at two hospitals, led to the discovery of elevated liver enzymes and subsequent referral to a tertiary care center. Clinical examination revealed hepatomegaly, abdominal distension, and non-palpable spleen. Laboratory findings confirmed acute hepatitis, prompting further investigation into the child's dietary history and revealing a potential foodborne infection. The child was diagnosed with hepatitis-associated severe vitamin A deficiency, manifested by Bitot's spots on ophthalmic examination. Prompt initiation of antiviral therapy, nutritional supplementation, and supportive care resulted in a positive clinical response, with resolution of symptoms and normalization of liver enzymes. This case underscores the importance of recognizing nutritional deficiencies in the context of infectious diseases, emphasizing the need for a comprehensive approach to patient care. The successful management of this complex case highlights the significance of interdisciplinary collaboration in ensuring optimal outcomes in pediatric patients with overlapping infectious and nutritional etiologies.

## Introduction

Hepatitis is a significant global health concern, particularly in pediatric populations, often presenting with a range of nonspecific symptoms, including fever, abdominal discomfort, and abnormal liver enzymes [1]. The etiology of hepatitis in children can vary, encompassing infectious, metabolic, and autoimmune causes [1]. While viral infections, such as hepatitis A and B, are well-recognized culprits, nutritional deficiencies may also contribute to liver dysfunction in children. Vitamin A, a fat-soluble micronutrient, is crucial for maintaining the integrity of epithelial tissues, immune function, and vision [2]. In regions with inadequate dietary intake and limited access to nutritional resources, vitamin A deficiency remains a prevalent issue, particularly among children [3]. The liver plays a central role in vitamin A metabolism, and deficiency can lead to clinical manifestations, including xerophthalmia and Bitot's spots. Furthermore, hepatic dysfunction may occur due to disrupted vitamin A metabolism [4].

The association between hepatitis and altered vitamin A metabolism has been explored in the literature. Studies suggest that viral hepatitis may disrupt the intricate balance of vitamin A homeostasis, potentially leading to deficiency [5]. Additionally, the liver's role in storing and releasing vitamin A makes it susceptible to dysfunction in the context of hepatic diseases [5]. The clinical presentation of pediatric patients with both hepatitis and severe vitamin A deficiency can be complex and challenging to diagnose. Hepatitis may overshadow the signs of nutritional deficiencies, leading to delayed identification and intervention [6]. Therefore, a comprehensive approach combining clinical history, physical examination, and specialized investigations is essential for accurate diagnosis and timely management.

In resource-limited settings, where access to safe and nutritious food may be constrained, waterborne infections can exacerbate nutritional deficiencies in children [7]. The potential synergistic effect of viral hepatitis and foodborne infections on liver function underscores the importance of considering both infectious and nutritional etiologies in the evaluation of pediatric hepatomegaly. Managing pediatric patients with hepatitis-induced severe vitamin A deficiency necessitates a multidisciplinary approach involving pediatricians, hepatologists, and ophthalmologists-early recognition and intervention, including direct-acting antivirals (DAAs) and host-targeting agents (HTAs). DAAs specifically target the hepatitis A virus (HAV) and include protease, polymerase, internal ribosome entry site (IRES), and nutritional supplementation, which are crucial for achieving positive outcomes [8]. Public health strategies focused on improving dietary practices and ensuring access to micronutrient-rich foods are pivotal in preventing nutritional deficiencies in at-risk populations [9].

## Case presentation

A three-year-old male child was brought to the casualty department with complaints of fever, abdominal distention, and loose stools. According to the parents, the child was in good health until five days ago when he developed a moderate-grade fever associated with chills and rigors. The fever, initially responsive to medications, persisted, prompting the parents to seek medical attention at a private hospital. After two days of oral medications without relief, the child developed sudden-onset abdominal distention, progressively increasing in size. The child's medical history revealed a lack of diurnal variation in fever, no relief with medication, and no history of previous blood transfusions. Routine vaccination was given to the patient as per the age. The parents sought care at another hospital, where elevated liver enzymes (serum glutamic oxaloacetic transaminase [SGOT] and serum glutamate pyruvate transaminase [SGPT]) were detected, as described in Table 1. Subsequently, the child was referred to our tertiary care center due to the presence of liver swelling.

**Table 1:** Laboratory values of patient

Variable	Patient Value	Normal Range
Serum Glutamic-Oxaloacetic Transaminase (SGOT)	291	10–40 IU/L
Serum Glutamic Pyruvic Transaminase (SGPT)	1190	7 to 56 U/L
Albumin	2.2	3.4 to 5.4 g/dL
Bilirubin	6.7	5.1-17 μmol/L
Hemoglobin	7.6	9.5–14.1 g/dL

For two days, the child also complained of loose stools, described as non-foul smelling. There was one episode of vomiting with water as the content. The parents reported decreased oral intake and constipation for the past two days. Notably, there was a history of the child consuming outside food. Upon arrival at our facility, the child's vital signs were as follows: heart rate 123 beats per minute, respiratory rate 26 breaths per minute, blood pressure 94/54 mmHg, and oxygen saturation 99%. Abdominal girth was measured at 53.5 cm. Systemic examination revealed uniform abdominal distension with full flanks, an everted umbilicus with no visible pulsation, and normal skin over the abdomen. Shifting dullness was present on percussion, and the liver was palpable 4 cm below the costal margin. The spleen was non-palpable.

Cardiovascular examination revealed normal S1S2 with no murmurs, respiratory examination showed equal bilateral air entry with no added sounds, and the central nervous system examination indicated a conscious, alert, and awake child. Laboratory investigations demonstrated elevated liver enzymes (SGOT and SGPT), consistent with acute hepatitis. Stool examination revealed non-foul-smelling loose stools. These findings and the child's history of outside food consumption raised concerns about a possible foodborne infection.

**Figure 1:**
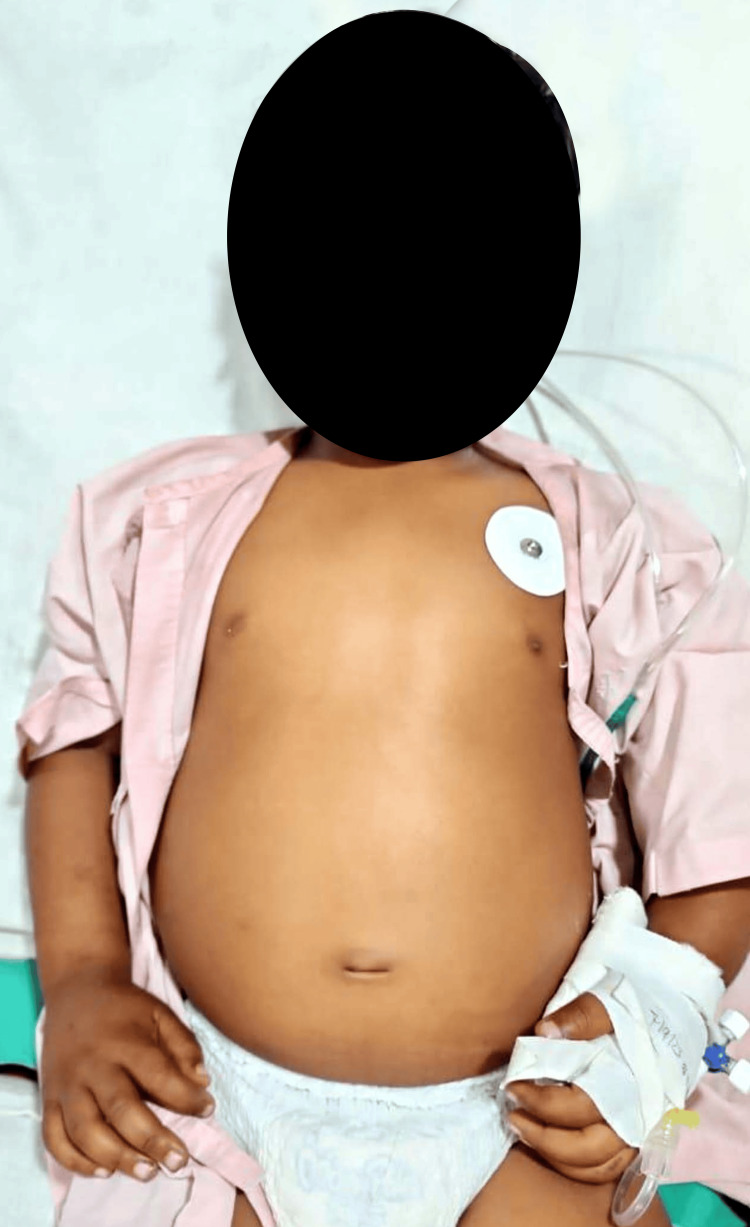
Shows distention of the abdomen

**Figure 2:**
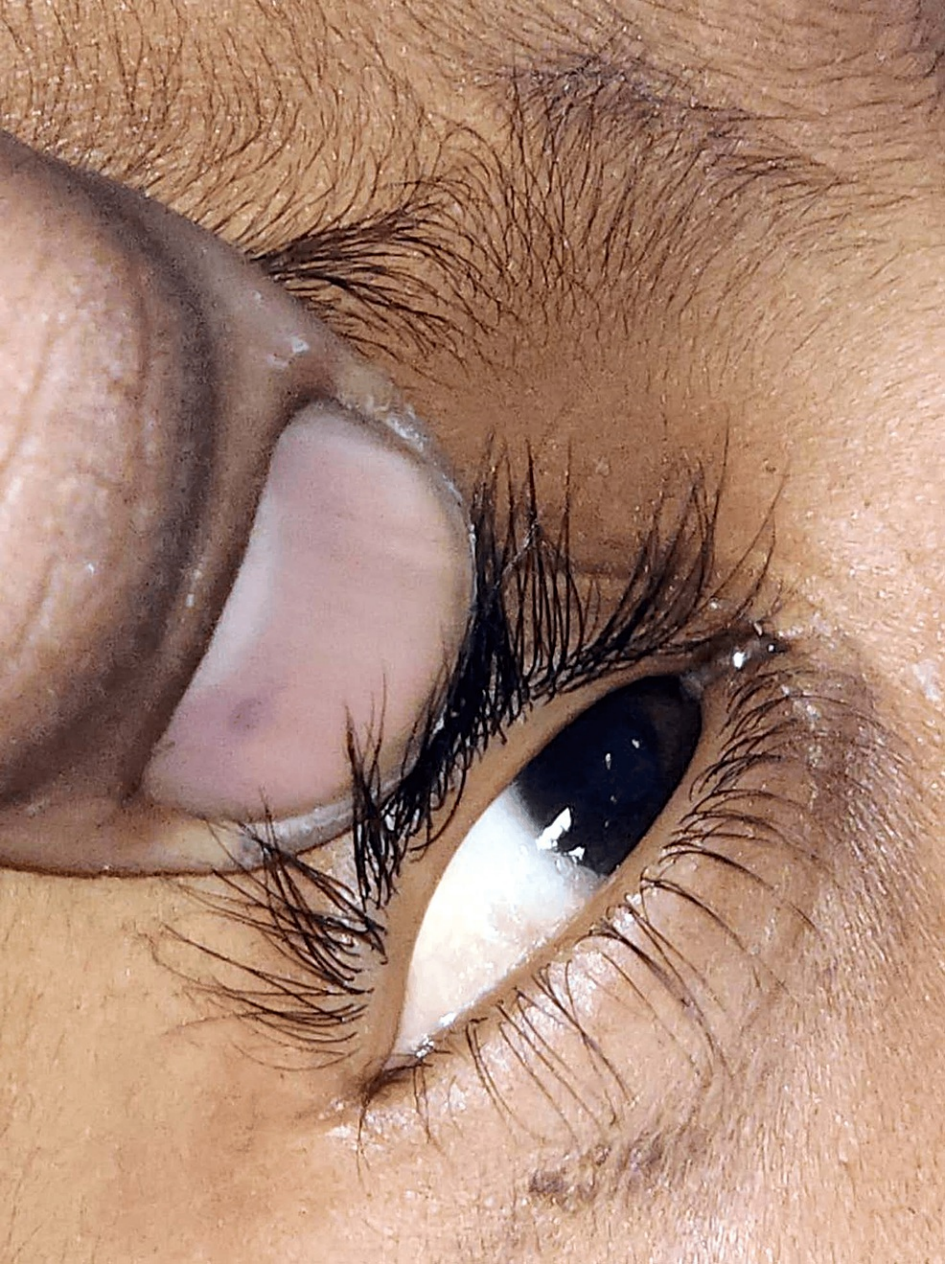
Shows Bitot's spots in the eyes

The child was diagnosed with hepatitis, leading to severe vitamin A deficiency. The abdominal distension and hepatomegaly were attributed to ascites secondary to liver dysfunction Figure 1. Bitot's spots, observed on ophthalmic examination Figure 2, confirmed the severe vitamin A deficiency. The patient was initiated on antiviral therapy for hepatitis, nutritional supplementation, including vitamin A, and supportive care. The child responded well to treatment, with a gradual resolution of symptoms and normalization of liver enzymes. Follow-up examinations revealed a significant improvement in Bitot's spots.

## Discussion

The presented case is the importance of a comprehensive approach to pediatric patients with fever, abdominal symptoms, and abnormal liver enzymes. The combination of clinical history, physical examination, and laboratory findings led to the diagnosis of hepatitis-A-induced severe vitamin A deficiency, emphasizing the need for clinicians to consider nutritional deficiencies in the differential diagnosis of hepatic disorders in children. Hepatitis is a common cause of liver dysfunction in children, often presenting with symptoms such as fever, abdominal distention, and abnormal liver enzymes [1]. In this case, the concurrent occurrence of severe vitamin A deficiency is notable, as vitamin A plays a crucial role in maintaining the integrity of epithelial surfaces, including those of the liver [3]. Previous studies have suggested a link between hepatitis and altered vitamin A metabolism, emphasizing the need to explore this association further [10].

The initial presentation of hepatitis without a clear etiology posed diagnostic challenges. The discovery of Bitot's spots on ophthalmic examination was pivotal in diagnosing severe vitamin A deficiency. Bitot's spots, characterized by foamy, irregular, elevated plaques on the conjunctiva, are pathognomonic for vitamin A deficiency [11]. Recognition of these clinical signs is crucial for early diagnosis and intervention. The history of outside food consumption raised concerns about a possible foodborne infection contributing to the hepatitic presentation. Foodborne infections, especially in resource-limited settings, can exacerbate nutritional deficiencies and complicate the clinical picture [7]. This case highlights the importance of obtaining a detailed dietary history in children with hepatomegaly.

The successful management of the presented case involved antiviral therapy for hepatitis, nutritional supplementation, and supportive care. The gradual resolution of symptoms, normalization of liver enzymes, and improvement in Bitot's spots underscore the importance of a multidisciplinary approach involving pediatricians, hepatologists, and ophthalmologists. This case highlights the potential role of preventive strategies in reducing the incidence of severe vitamin A deficiency in children. Public health interventions, including dietary supplementation programs and health education, could contribute to the prevention of nutritional deficiencies in at-risk populations [12].

## Conclusions

In conclusion, timely identification, precise diagnosis, and effective intervention are pivotal in achieving favorable outcomes for cases involving severe vitamin A deficiency induced by hepatitis. While this report adds valuable insights to the expanding realm of knowledge concerning the varied presentations of pediatric hepatobiliary disorders, it is acknowledged that the conclusion could benefit from a more comprehensive approach. Emphasizing the significance of a holistic and multidisciplinary strategy in pediatric healthcare, it is essential to underscore the necessity for long-term follow-up and sustained research efforts. These endeavors are crucial for gaining a deeper understanding of the intricate interconnections between these conditions and refining strategies aimed at prevention and management.
